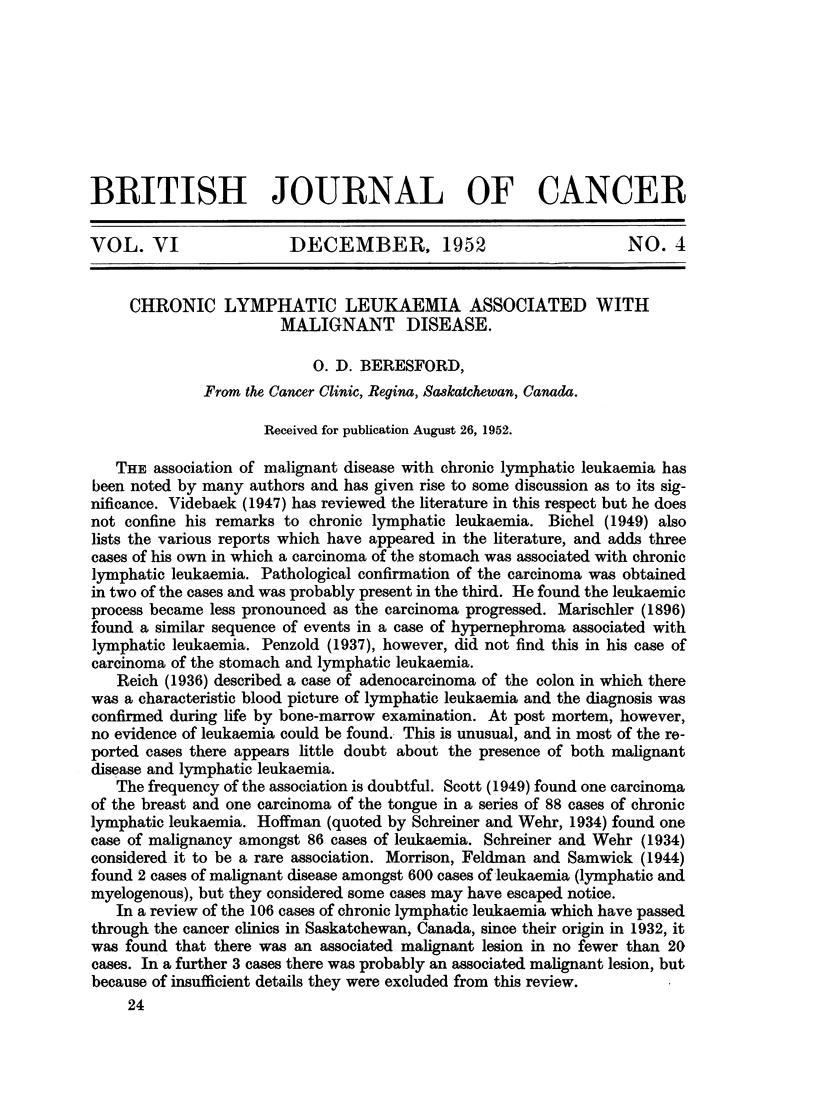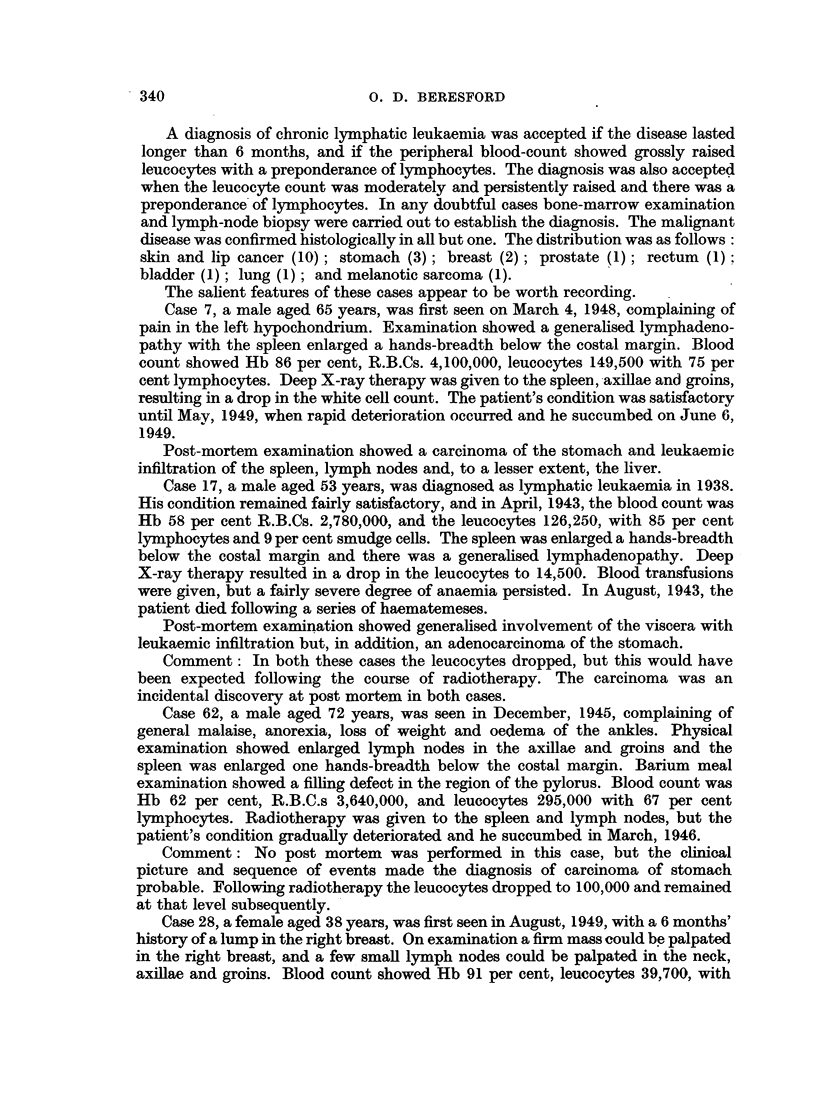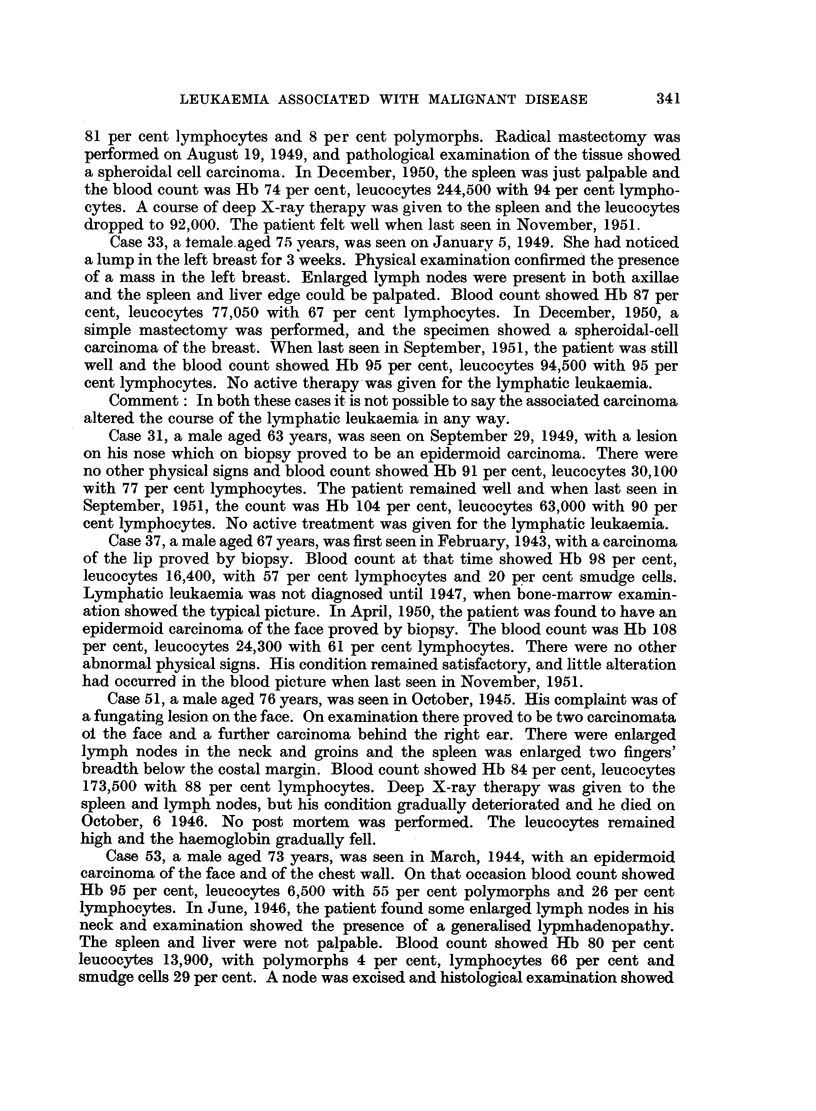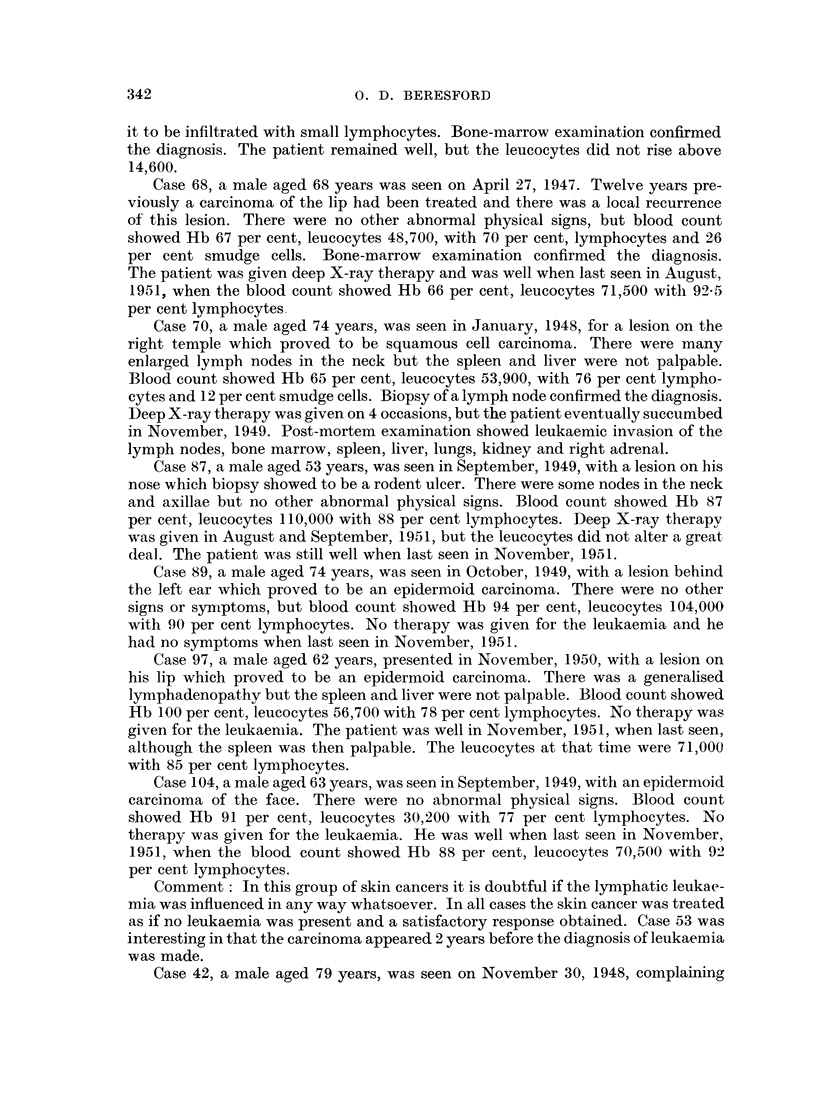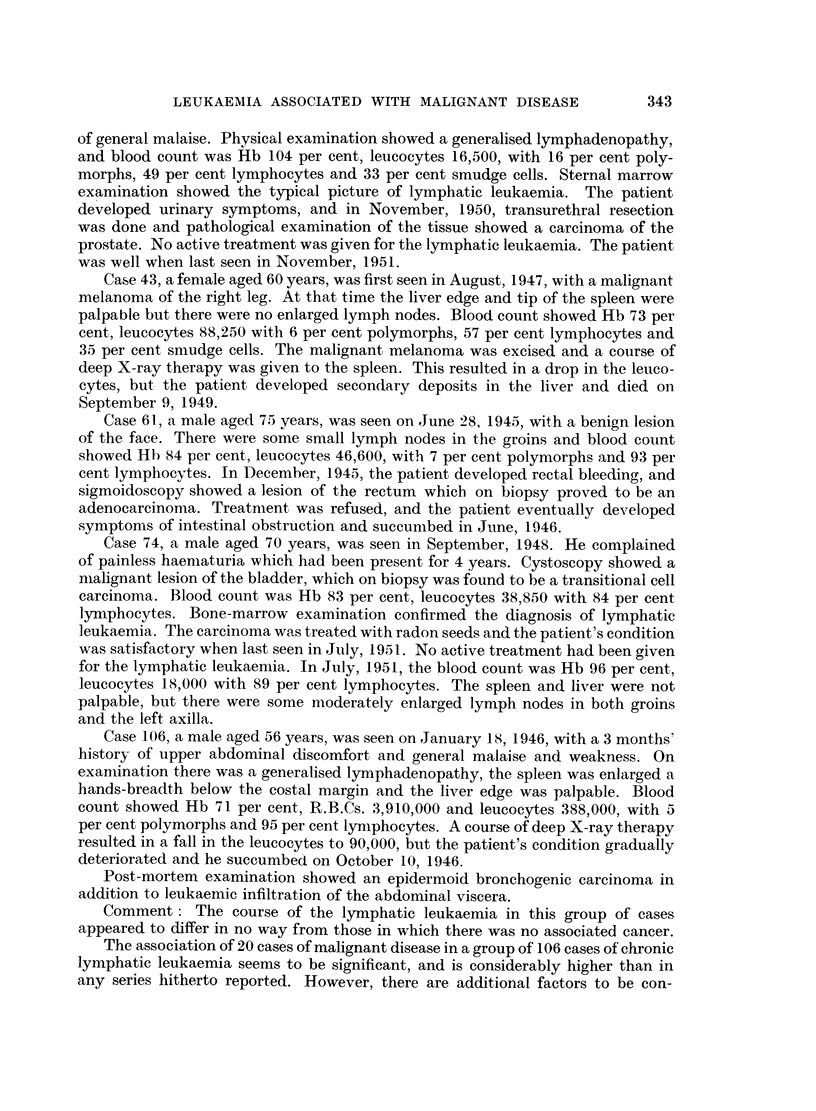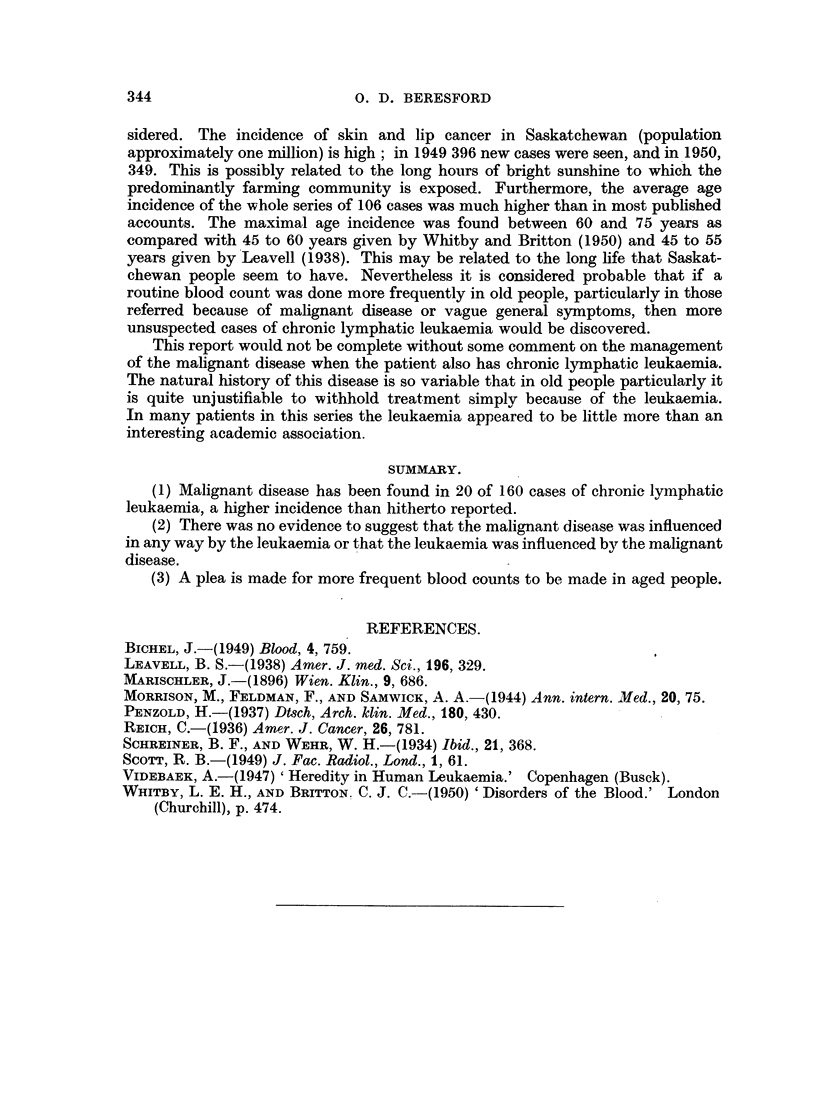# Chronic Lymphatic Leukaemia Associated with Malignant Disease

**DOI:** 10.1038/bjc.1952.37

**Published:** 1952-12

**Authors:** O. D. Beresford


BRITISH JOURNAL OF CANCER

VOL. VI DECEMBER, 1952 NO. 4

### CHRONIC LYMPHATIC LEUKAEMIA ASSOCIATED WITH MALIGNANT DISEASE.

O. D. BERESFORD,

*From the Cancer Clinic, Regina, Saskatchewan, Canada.*

Received for publication August 26, 1952.

THE association of malignant disease with chronic lymphatic leukaemia has been noted by many authors and has given rise to some discussion as to its significance. Videbaek (1947) has reviewed the literature in this respect but he does not confine his remarks to chronic lymphatic leukaemia. Bichel (1949) also lists the various reports which have appeared in the literature, and adds three cases of his own in which a carcinoma of the stomach was associated with chronic lymphatic leukaemia. Pathological confirmation of the carcinoma was obtained in two of the cases and was probably present in the third. He found the leukaemic process became less pronounced as the carcinoma progressed. Marischler (1896) found a similar sequence of events in a case of hypernephroma associated with lymphatic leukaemia. Penzold (1937), however, did not find this in his case of carcinoma of the stomach and lymphatic leukaemia.

Reich (1936) described a case of adenocarcinoma of the colon in which there was a characteristic blood picture of lymphatic leukaemia and the diagnosis was confirmed during life by bone-marrow examination. At post mortem, however, no evidence of leukaemia could be found. This is unusual, and in most of the reported cases there appears little doubt about the presence of both malignant disease and lymphatic leukaemia.

The frequency of the association is doubtful. Scott (1949) found one carcinoma of the breast and one carcinoma of the tongue in a series of 88 cases of chronic lymphatic leukaemia. Hoffman (quoted by Schreiner and Wehr, 1934) found one case of malignancy amongst 86 cases of leukaemia. Schreiner and Wehr (1934) considered it to be a rare association. Morrison, Feldman and Samwick (1944) found 2 cases of malignant disease amongst 600 cases of leukaemia (lymphatic and myelogenous), but they considered some cases may have escaped notice.

In a review of the 106 cases of chronic lymphatic leukaemia which have passed through the cancer clinics in Saskatchewan, Canada, since their origin in 1932, it was found that there was an associated malignant lesion in no fewer than 20 cases. In a further 3 cases there was probably an associated malignant lesion, but because of insufficient details they were excluded from this review.

A diagnosis of chronic lymphatic leukaemia was accepted if the disease lasted longer than 6 months, and if the peripheral blood-count showed grossly raised leucocytes with a preponderance of lymphocytes. The diagnosis was also accepted when the leucocyte count was moderately and persistently raised and there was a preponderance of lymphocytes. In any doubtful cases bone-marrow examination and lymph-node biopsy were carried out to establish the diagnosis. The malignant disease was confirmed histologically in all but one. The distribution was as follows : skin and lip cancer (10) ; stomach (3) ; breast (2) ; prostate (1) ; rectum (1) ; bladder (1) ; lung (1) ; and melanotic sarcoma (1).

The salient features of these cases appear to be worth recording.

Case 7, a male aged 65 years, was first seen on March 4, 1948, complaining of pain in the left hypochondrium. Examination showed a generalised lymphadenopathy with the spleen enlarged a hands-breadth below the costal margin. Blood count showed Hb 86 per cent, R.B.Cs. 4,100,000, leucocytes 149,500 with 75 per cent lymphocytes. Deep X-ray therapy was given to the spleen, axillae and groins, resulting in a drop in the white cell count. The patient's condition was satisfactory until May, 1949, when rapid deterioration occurred and he succumbed on June 6, 1949.

Post-mortem examination showed a carcinoma of the stomach and leukaemic infiltration of the spleen, lymph nodes and, to a lesser extent, the liver.

Case 17, a male aged 53 years, was diagnosed as lymphatic leukaemia in 1938. His condition remained fairly satisfactory, and in April, 1943, the blood count was Hb 58 per cent R.B.Cs. 2,780,000, and the leucocytes 126,250, with 85 per cent lymphocytes and 9 per cent smudge cells. The spleen was enlarged a hands-breadth below the costal margin and there was a generalised lymphadenopathy. Deep X-ray therapy resulted in a drop in the leucocytes to 14,500. Blood transfusions were given, but a fairly severe degree of anaemia persisted. In August, 1943, the patient died following a series of haematemeses.

Post-mortem examination showed generalised involvement of the viscera with leukaemic infiltration but, in addition, an adenocarcinoma of the stomach.

Comment : In both these cases the leucocytes dropped, but this would have been expected following the course of radiotherapy. The carcinoma was an incidental discovery at post mortem in both cases.

Case 62, a male aged 72 years, was seen in December, 1945, complaining of general malaise, anorexia, loss of weight and oedema of the ankles. Physical examination showed enlarged lymph nodes in the axillae and groins and the spleen was enlarged one hands-breadth below the costal margin. Barium meal examination showed a filling defect in the region of the pylorus. Blood count was Hb 62 per cent, R.B.C.s 3,640,000, and leucocytes 295,000 with 67 per cent lymphocytes. Radiotherapy was given to the spleen and lymph nodes, but the patient's condition gradually deteriorated and he succumbed in March, 1946.

Comment : No post mortem was performed in this case, but the clinical picture and sequence of events made the diagnosis of carcinoma of stomach probable. Following radiotherapy the leucocytes dropped to 100,000 and remained at that level subsequently.

Case 28, a female aged 38 years, was first seen in August, 1949, with a 6 months' history of a lump in the right breast. On examination a firm mass could be palpated in the right breast, and a few small lymph nodes could be palpated in the neck, axillae and groins. Blood count showed Hb 91 per cent, leucocytes 39,700, with

81 per cent lymphocytes and 8 per cent polymorphs. Radical mastectomy was performed on August 19, 1949, and pathological examination of the tissue showed a spheroidal cell carcinoma. In December, 1950, the spleen was just palpable and the blood count was Hb 74 per cent, leucocytes 244,500 with 94 per cent lymphocytes. A course of deep X-ray therapy was given to the spleen and the leucocytes dropped to 92,000. The patient felt well when last seen in November, 1951.

Case 33, a female aged 75 years, was seen on January 5, 1949. She had noticed a lump in the left breast for 3 weeks. Physical examination confirmed the presence of a mass in the left breast. Enlarged lymph nodes were present in both axillae and the spleen and liver edge could be palpated. Blood count showed Hb 87 per cent, leucocytes 77,050 with 67 per cent lymphocytes. In December, 1950, a simple mastectomy was performed, and the specimen showed a spheroidal-cell carcinoma of the breast. When last seen in September, 1951, the patient was still well and the blood count showed Hb 95 per cent, leucocytes 94,500 with 95 per cent lymphocytes. No active therapy was given for the lymphatic leukaemia.

Comment : In both these cases it is not possible to say the associated carcinoma altered the course of the lymphatic leukaemia in any way.

Case 31, a male aged 63 years, was seen on September 29, 1949, with a lesion on his nose which on biopsy proved to be an epidermoid carcinoma. There were no other physical signs and blood count showed Hb 91 per cent, leucocytes 30,100 with 77 per cent lymphocytes. The patient remained well and when last seen in September, 1951, the count was Hb 104 per cent, leucocytes 63,000 with 90 per cent lymphocytes. No active treatment was given for the lymphatic leukaemia.

Case 37, a male aged 67 years, was first seen in February, 1943, with a carcinoma of the lip proved by biopsy. Blood count at that time showed Hb 98 per cent, leucocytes 16,400, with 57 per cent lymphocytes and 20 per cent smudge cells. Lymphatic leukaemia was not diagnosed until 1947, when bone-marrow examination showed the typical picture. In April, 1950, the patient was found to have an epidermoid carcinoma of the face proved by biopsy. The blood count was Hb 108 per cent, leucocytes 24,300 with 61 per cent lymphocytes. There were no other abnormal physical signs. His condition remained satisfactory, and little alteration had occurred in the blood picture when last seen in November, 1951.

Case 51, a male aged 76 years, was seen in October, 1945. His complaint was of a fungating lesion on the face. On examination there proved to be two carcinomata of the face and a further carcinoma behind the right ear. There were enlarged lymph nodes in the neck and groins and the spleen was enlarged two fingers' breadth below the costal margin. Blood count showed Hb 84 per cent, leucocytes 173,500 with 88 per cent lymphocytes. Deep X-ray therapy was given to the spleen and lymph nodes, but his condition gradually deteriorated and he died on October, 6 1946. No post mortem was performed. The leucocytes remained high and the haemoglobin gradually fell.

Case 53, a male aged 73 years, was seen in March, 1944, with an epidermoid carcinoma of the face and of the chest wall. On that occasion blood count showed Hb 95 per cent, leucocytes 6,500 with 55 per cent polymorphs and 26 per cent lymphocytes. In June, 1946, the patient found some enlarged lymph nodes in his neck and examination showed the presence of a generalised lypmhadenopathy. The spleen and liver were not palpable. Blood count showed Hb 80 per cent leucocytes 13,900, with polymorphs 4 per cent, lymphocytes 66 per cent and smudge cells 29 per cent. A node was excised and histological examination showed it to be infiltrated with small lymphocytes. Bone-marrow examination confirmed the diagnosis. The patient remained well, but the leucocytes did not rise above 14,600.

Case 68, a male aged 68 years was seen on April 27, 1947. Twelve years previously a carcinoma of the lip had been treated and there was a local recurrence of this lesion. There were no other abnormal physical signs, but blood count showed Hb 67 per cent, leucocytes 48,700, with 70 per cent, lymphocytes and 26 per cent smudge cells. Bone-marrow examination confirmed the diagnosis. The patient was given deep X-ray therapy and was well when last seen in August, 1951, when the blood count showed Hb 66 per cent, leucocytes 71,500 with 92·5 per cent lymphocytes.

Case 70, a male aged 74 years, was seen in January, 1948, for a lesion on the right temple which proved to be squamous cell carcinoma. There were many enlarged lymph nodes in the neck but the spleen and liver were not palpable. Blood count showed Hb 65 per cent, leucocytes 53,900, with 76 per cent lymphocytes and 12 per cent smudge cells. Biopsy of a lymph node confirmed the diagnosis. Deep X-ray therapy was given on 4 occasions, but the patient eventually succumbed in November, 1949. Post-mortem examination showed leukaemic invasion of the lymph nodes, bone marrow, spleen, liver, lungs, kidney and right adrenal.

Case 87, a male aged 53 years, was seen in September, 1949, with a lesion on his nose which biopsy showed to be a rodent ulcer. There were some nodes in the neck and axillae but no other abnormal physical signs. Blood count showed Hb 87 per cent, leucocytes 110,000 with 88 per cent lymphocytes. Deep X-ray therapy was given in August and September, 1951, but the leucocytes did not alter a great deal. The patient was still well when last seen in November, 1951.

Case 89, a male aged 74 years, was seen in October, 1949, with a lesion behind the left ear which proved to be an epidermoid carcinoma. There were no other signs or symptoms, but blood count showed Hb 94 per cent, leucocytes 104,000 with 90 per cent lymphocytes. No therapy was given for the leukaemia and he had no symptoms when last seen in November, 1951.

Case 97, a male aged 62 years, presented in November, 1950, with a lesion on his lip which proved to be an epidermoid carcinoma. There was a generalised lymphadenopathy but the spleen and liver were not palpable. Blood count showed Hb 100 per cent, leucocytes 56,700 with 78 per cent lymphocytes. No therapy was given for the leukaemia. The patient was well in November, 1951, when last seen, although the spleen was then palpable. The leucocytes at that time were 71,000 with 85 per cent lymphocytes.

Case 104, a male aged 63 years, was seen in September, 1949, with an epidermoid carcinoma of the face. There were no abnormal physical signs. Blood count showed Hb 91 per cent, leucocytes 30,200 with 77 per cent lymphocytes. No therapy was given for the leukaemia. He was well when last seen in November, 1951, when the blood count showed Hb 88 per cent, leucocytes 70,500 with 92 per cent lymphocytes.

Comment : In this group of skin cancers it is doubtful if the lymphatic leukaemia was influenced in any way whatsoever. In all cases the skin cancer was treated as if no leukaemia was present and a satisfactory response obtained. Case 53 was interesting in that the carcinoma appeared 2 years before the diagnosis of leukaemia was made.

Case 42, a male aged 79 years, was seen on November 30, 1948, complaining of general malaise. Physical examination showed a generalised lymphadenopathy, and blood count was Hb 104 per cent, leucocytes 16,500, with 16 per cent polymorphs, 49 per cent lymphocytes and 33 per cent smudge cells. Sternal marrow examination showed the typical picture of lymphatic leukaemia. The patient developed urinary symptoms, and in November, 1950, transurethral resection was done and pathological examination of the tissue showed a carcinoma of the prostate. No active treatment was given for the lymphatic leukaemia. The patient was well when last seen in November, 1951.

Case 43, a female aged 60 years, was first seen in August, 1947, with a malignant melanoma of the right leg. At that time the liver edge and tip of the spleen were palpable but there were no enlarged lymph nodes. Blood count showed Hb 73 per cent, leucocytes 88,250 with 6 per cent polymorphs, 57 per cent lymphocytes and 35 per cent smudge cells. The malignant melanoma was excised and a course of deep X-ray therapy was given to the spleen. This resulted in a drop in the leucocytes, but the patient developed secondary deposits in the liver and died on September 9, 1949.

Case 61, a male aged 75 years, was seen on June 28, 1945, with a benign lesion of the face. There were some small lymph nodes in the groins and blood count showed Hb 84 per cent, leucocytes 46,600, with 7 per cent polymorphs and 93 per cent lymphocytes. In December, 1945, the patient developed rectal bleeding, and sigmoidoscopy showed a lesion of the rectum which on biopsy proved to be an adenocarcinoma. Treatment was refused, and the patient eventually developed symptoms of intestinal obstruction and succumbed in June, 1946.

Case 74, a male aged 70 years, was seen in September, 1948. He complained of painless haematuria which had been present for 4 years. Cystoscopy showed a malignant lesion of the bladder, which on biopsy was found to be a transitional cell carcinoma. Blood count was Hb 83 per cent, leucocytes 38,850 with 84 per cent lymphocytes. Bone-marrow examination confirmed the diagnosis of lymphatic leukaemia. The carcinoma was treated with radon seeds and the patient's condition was satisfactory when last seen in July, 1951. No active treatment had been given for the lymphatic leukaemia. In July, 1951, the blood count was Hb 96 per cent, leucocytes 18,000 with 89 per cent lymphocytes. The spleen and liver were not palpable, but there were some moderately enlarged lymph nodes in both groins and the left axilla.

Case 106, a male aged 56 years, was seen on January 18, 1946, with a 3 months' history of upper abdominal discomfort and general malaise and weakness. On examination there was a generalised lymphadenopathy, the spleen was enlarged a hands-breadth below the costal margin and the liver edge was palpable. Blood count showed Hb 71 per cent, R.B.Cs. 3,910,000 and leucocytes 388,000, with 5 per cent polymorphs and 95 per cent lymphocytes. A course of deep X-ray therapy resulted in a fall in the leucocytes to 90,000, but the patient's condition gradually deteriorated and he succumbed on October 10, 1946.

Post-mortem examination showed an epidermoid bronchogenic carcinoma in addition to leukaemic infiltration of the abdominal viscera.

Comment : The course of the lymphatic leukaemia in this group of cases appeared to differ in no way from those in which there was no associated cancer.

The association of 20 cases of malignant disease in a group of 106 cases of chronic lymphatic leukaemia seems to be significant, and is considerably higher than in any series hitherto reported. However, there are additional factors to be considered. The incidence of skin and lip cancer in Saskatchewan (population approximately one million) is high ; in 1949 396 new cases were seen, and in 1950, 349. This is possibly related to the long hours of bright sunshine to which the predominantly farming community is exposed. Furthermore, the average age incidence of the whole series of 106 cases was much higher than in most published accounts. The maximal age incidence was found between 60 and 75 years as compared with 45 to 60 years given by Whitby and Britton (1950) and 45 to 55 years given by Leavell (1938). This may be related to the long life that Saskatchewan people seem to have. Nevertheless it is considered probable that if a routine blood count was done more frequently in old people, particularly in those referred because of malignant disease or vague general symptoms, then more unsuspected cases of chronic lymphatic leukaemia would be discovered.

This report would not be complete without some comment on the management of the malignant disease when the patient also has chronic lymphatic leukaemia. The natural history of this disease is so variable that in old people particularly it is quite unjustifiable to withhold treatment simply because of the leukaemia. In many patients in this series the leukaemia appeared to be little more than an interesting academic association.

### SUMMARY.

(1) Malignant disease has been found in 20 of 160 cases of chronic lymphatic leukaemia, a higher incidence than hitherto reported.

(2) There was no evidence to suggest that the malignant disease was influenced in any way by the leukaemia or that the leukaemia was influenced by the malignant disease.

(3) A plea is made for more frequent blood counts to be made in aged people.

### REFERENCES.

Bichel, J.—(1949) *Blood*, **4**, 759.
Leavell, B. S.—(1938) *Amer. J. med. Sci.*, **196**, 329.
Marischler, J.—(1896) *Wien. Klin.*, **9**, 686.
Morrison, M., Feldman, F., and Samwick, A. A.—(1944) *Ann. intern. Med.*, **20**, 75.
Penzold, H.—(1937) *Dtsch, Arch. klin. Med.*, **180**, 430.
Reich, C.—(1936) *Amer. J. Cancer*, **26**, 781.
Schreiner, B. F., and Wehr, W. H.—(1934) *Ibid.*, **21**, 368.
Scott, R. B.—(1949) *J. Fac. Radiol., Lond.*, **1**, 61.
Videbaek, A.—(1947) 'Heredity in Human Leukaemia.' Copenhagen (Busck).
Whitby, L. E. H., and Britton, C. J. C.—(1950) 'Disorders of the Blood.' London (Churchill), p. 474.